# Effects of dietary *omega*-3 polyunsaturated fatty acids on growth and immune response of weanling pigs

**DOI:** 10.1186/2055-0391-56-7

**Published:** 2014-07-24

**Authors:** Qizhang Li, Joel H Brendemuhl, Kwang C Jeong, Lokenga Badinga

**Affiliations:** Department of Animal Sciences, Institute of Food and Agricultural Sciences, University of Florida, Gainesville, 32611 USA

**Keywords:** n-3 PUFA, Growth, Immunity, Pig

## Abstract

The recognition that *omega*-3 polyunsaturated fatty acids (*n*-3 PUFA) possess potent anti-inflammatory properties in human models has prompted studies investigating their efficacy for animal growth and immunity. This study examined the effect of feeding an *n*-3 PUFA-enriched diet on growth and immune response of weanling piglets. Newly weaned pigs (averaging 27 ± 2 days of age and 8.1 ± 0.7 kg of body weight) were assigned randomly to receive a control (3% vegetable oil, n = 20) or *n*-3 PUFA-supplemented (3% marine *n*-3 PUFA, n = 20) diet for 28 day after weaning. Female pigs consuming the *n*-3 PUFA-enriched diet were lighter at week 4 post-weaning than those fed the vegetable oil supplement. Weanling pigs gained more weight, consumed more feed and had better growth to feed ratios between days 14 and 28 than between days 0 and 14 post-weaning. Plasma insulin-like growth factor I (IGF-I) decreased between days 0 (87.2 ± 17.0 ng/mL) and 14 (68.3 ± 21.1 ng/mL) after weaning and then increased again by day 28 (155.2 ± 20.9 ng/mL). In piglets consuming the vegetable oil-enriched diet, plasma tumor necrosis factor alpha (TNF-α) increased from 37.6 ± 14.5 to 102.9 ± 16.6 pg/mL between days 0 and 14 post-weaning and remained high through day 28 (99.0 ± 17.2 pg/mL). The TNF-α increase detected in the piglets fed vegetable oil was not observed in the piglets fed *n*-3 PUFA. Results indicate that weaning induces considerable immune stress in piglets and that this stress can be mitigated by dietary supplementation of *n*-3 PUFA.

## Background

Nutritional, environmental and immune challenges associated with weaning may lead to considerable economic losses to pork producers. This period is generally characterized by decreased voluntary feed intake, altered gut integrity and increased concentrations of inflammatory cytokines in blood [1–3]. These nutritional and physiological abnormalities often result in diarrhea and depression of growth in newly weaned piglets. Restrictions of antibiotic usage in swine have compelled the industry to find alternatives that offer both performance enhancement and protection from disease [4, 5]. In this regard, Liu et al. [6] reported that dietary fish oil reduced the release of pro-inflammatory cytokines in weaned pigs challenged with *Escherichia coli* lipopolysaccharide. A more recent study indicated that prenatal exposure to long-chain *n*-3 PUFA increased postnatal glucose absorption in piglets [7]. Although exact mechanisms by which dietary *n*-3 PUFA modulate immune and metabolic functions in pigs are yet to be fully elucidated, the above study would indicate that dietary *n*-3 PUFA may help the piglets adapt quickly to the rapidly changing diet at weaning [7].

Currently, there is very little information regarding the use of *n*-3 PUFA in the diets of pigs raised under minimal disease and stress conditions. To test the hypothesis that nutritional management strategies that attenuate intestinal inflammation may partition nutrients to skeletal muscle for optimal growth, this study was designed to examine the effects of dietary *n*-3 PUFA on growth and immune response of weanling pigs raised without an added bacterial or environmental challenge.

## Results and discussion

**Figure 1:**
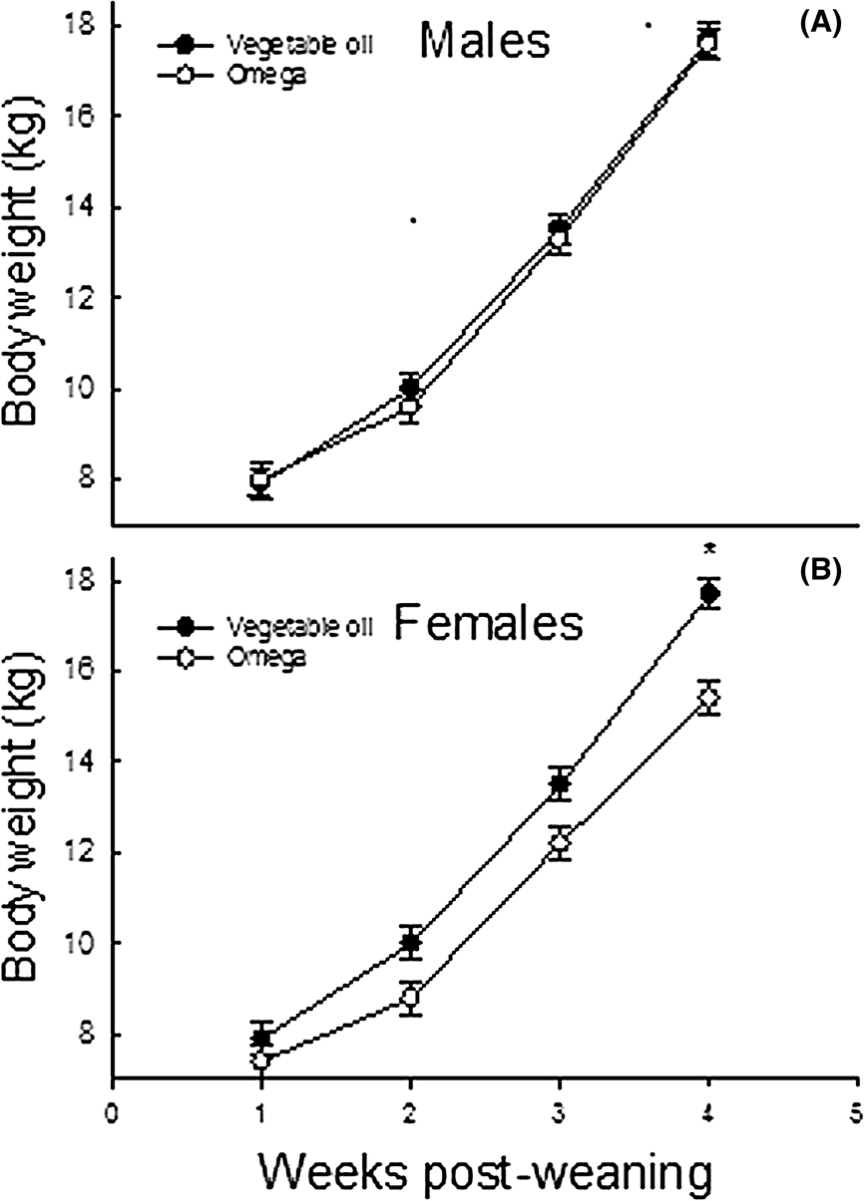
**Body weights of male (A) and female (B) pigs during four weeks after weaning.** A diet x gender x week interaction was detected (*P* < 0.04) for body weight. Asterisk indicates significant difference (*P* < 0.01) at the specified week.

Weaning imposes tremendous stress on piglets and is accompanied by marked changes in gastrointestinal physiology, microbiology and immunology [8]. The biochemical and histological changes that occur in the small intestine cause excessive secretion of pro-inflammatory cytokines and induce severe intestinal inflammation. *Omega*-3 PUFA are known to possess anti-inflammatory properties in humans [9, 10], swine [6, 11] and chickens [12]. To test the hypothesis that nutritional management strategies that attenuate intestinal inflammation may repartition nutrients to tissue accretion, we examined the effects of dietary *n*-3 PUFA on growth and immune response of weanling pigs (Figure 1) raised without an added bacterial or environmental challenge.

**Figure 2:**
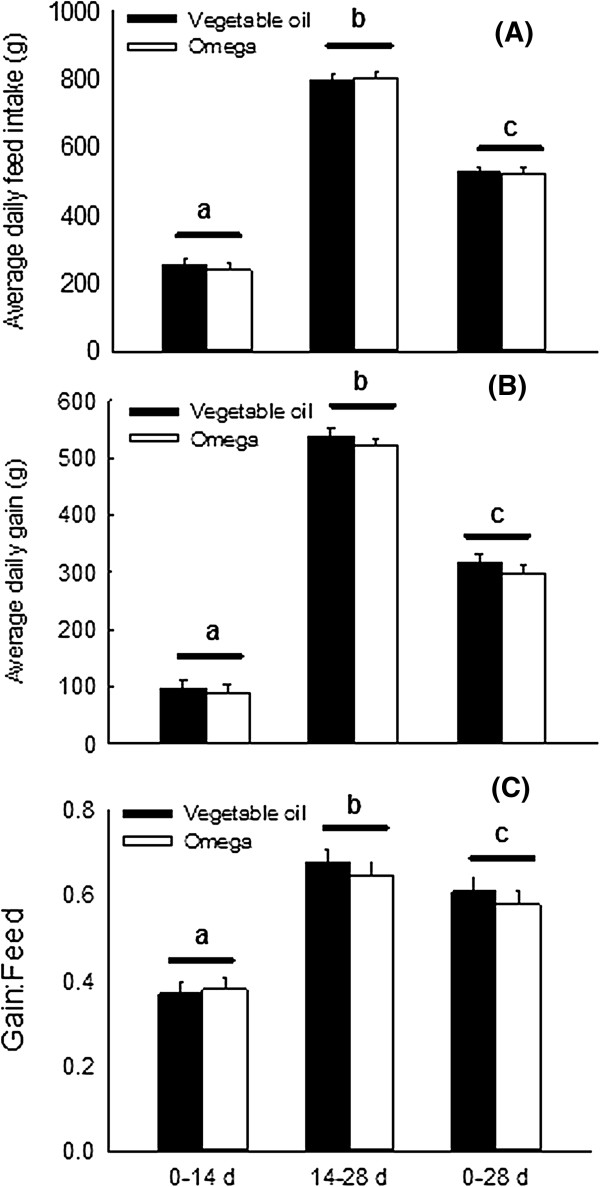
**Average daily feed intake (A), gain (B) and G: F (C) of weanling piglets fed diets with vegetable oil (Control, n = 20) or** ***n*** **-3 PUFA (Omega, n = 20).** For each response, pairs of histograms with different superscripts are different at *P* < 0.01. There were no differences among responses (P > 0.05) due to the dietary treatment.

Inclusion of 3% *n*-3 PUFA in the weanling piglet’s diet did not result in significantly improvement of average daily gain (ADG), average daily feed intake (ADFI) or growth to feed ratio (G: F) in weanling pigs. These findings are consistent with an earlier study [13] which detected no effects of dietary flax seed meal (rich in alpha linolenic acid) on basal body weight gain, feed intake or feed efficiency in weanling pigs. Additional studies using control diets with less 18: 3*n*-3 are needed to examine true effects of long-chain *n*-3 PUFA on growth and feed intake responses. Female piglets consuming the *n*-3 PUFA-supplemented diet were lighter at week 4 post-weaning than those consuming the vegetable oil-enriched diet. Whether or not this phenomenon was due to alteration in body composition as a result of feeding *n*-3 PUFA to nursery pigs was not documented. In rodents [14–16] and humans [17, 18], diets rich in *n*-3 PUFA lower fat stores and increase lean tissue mass. It is, therefore, possible that the smaller body weight of female piglets consuming *n*-3 PUFA detected at week 4 post-weaning was due to a decrease in fat accretion at the expense of lean tissue. The improvement of body weight gain detected in experimental animals between days 14 and 28 post-weaning likely resulted from an increase in feed intake and a decrease in basal inflammatory challenges during the second phase of growth (Figure 2).

**Figure 3:**
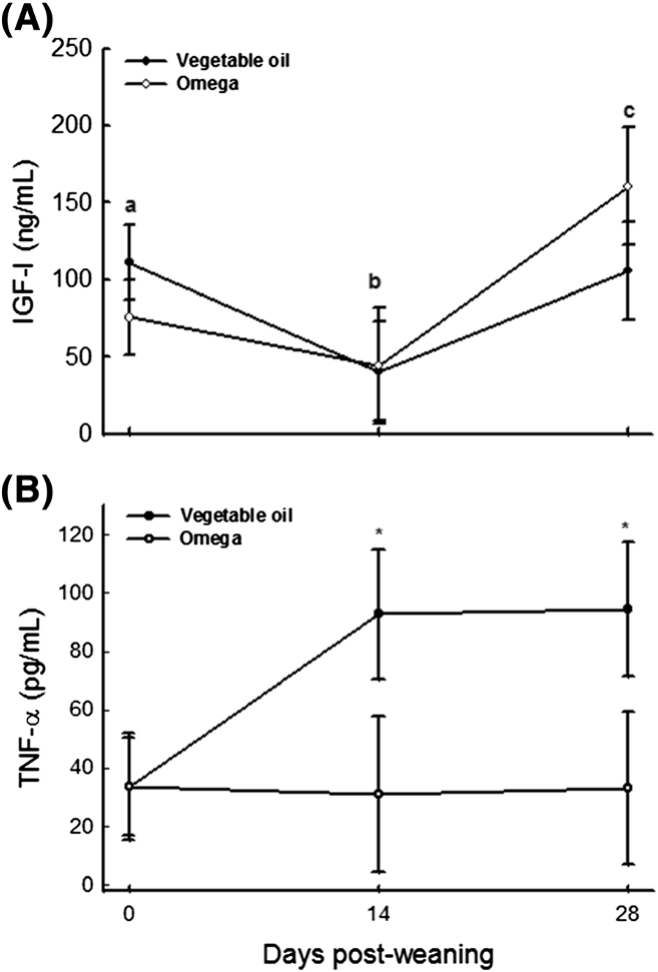
**Concentrations of IGF-I (A) and TNF-α (B) in plasma of weanling piglets fed diets with vegetable oil (Control, n = 20) or** ***n*** **-3 PUFA (** ***Omega*** **, n = 20).** Plasma IGF-I concentrations were affected by the growth phase (*P* < 0.01), but not the dietary treatment (*P* > 0.44). Plasma TNF-α concentrations were affected by the dietary treatment (*P* < 0.01), but not the growth phase (*P* > 0.28).

Concentrations of IGF-I in plasma decreased immediately following weaning and increased again by day 28 post-weaning (Figure 3). These findings indicate that weaning may cause a significant metabolic stress in weanling pigs and that this stress decreases with increasing weeks after weaning. There is little information on the effect of dietary *n*-3 PUFA on peripheral concentrations of growth factors in the pig. In the present study, inclusion of 3% *n*-3 PUFA into the piglet’s diet had no detectable effects on plasma IGF-I concentration during the first four weeks after weaning. These observations are consistent with previous studies [6, 13] which showed no beneficial effects of dietary fish oil on basal IGF-I concentration in weaned pigs. Thissen and Verniers [19] reported that IL-6 and TNF-α decreased both growth hormone (GH) and IGF-I mRNA in rat hepatocyte primary cultures. We did not examine GH or IGF-I transcript modulation by inflammatory cytokines, and therefore, whether or not the lack of *n*-3 PUFA effects on plasma IGF-I concentration detected in the present study was indicative of cytokine-mediated uncoupling of GH and IGF-I gene expression in weanling pigs warrants further investigation.

Tumor necrosis factor-α, a cytokine produced primarily by monocytes and macrophages, is thought to be one of the principal mediators of inflammation [20]. In the present study, plasma TNF-α concentrations were lower in weanling piglets supplemented with *n*-3 PUFA than those fed the vegetable oil supplement (Figure 3). These findings are consistent with previous in vitro [21–23] and in vivo [11, 24, 25] studies and suggest that *n*-3 PUFA inclusion in the diet could mitigate the immune stress in weanling pigs. Whereas exact mechanisms of *n*-3 PUFA suppression of TNF-α are yet to be fully elucidated, we speculate that suppression of TNF- α production by *n*-3 PUFA may be attributed, in part, to their inhibitory effects on NF-κB activation and or translocation to the nucleus [9, 22, 23]. Nuclear factor-κB are normally confined in the cytoplasm through their association with IκΒ. When cells are activated by inflammatory stimuli, the IκΒ are rapidly phosphorylated and degraded to free the NF-κΒ. The free NF-κΒ then migrate to the nucleus where they bind to cognate DNA binding sites and activate inflammatory gene transcription [9]. Any factor that prevents IκΒ phosphorylation and, thus, NF-κΒ activation, will decrease pro-inflammatory gene expression in the nucleus. Additionally, long-chain PUFA serve as ligands for peroxisome proliferator-activated receptors (PPAR), which are known to inhibit nuclear translocation of NF-κΒ [9]. Thus, activation of PPAR may be another intracellular mechanism by which marine *n*-3 PUFA regulate NF-κΒ activation and TNF-α production in animal models [9].

Hematological traits of swine are influenced by a variety of environmental and physiological factors including diet, age, gender and housing [26, 27]. In the present study, most of the blood characteristics examined did not differ among pigs fed the two diets (Table 1). Blood samples for complete blood cell counts were collected at 4 weeks after weaning, and it is possible that by this sampling time, the weanling piglets had already recovered from most physiological and dietary challenges normally associated with weaning in pigs. Alternatively, the piglets used in this study were raised in a clean environment and, thus, may not have acquired the “normal” gastrointestinal microflora that would cause clinical diseases. This hypothesis was further supported by our inability to detect salmonella and enterotoxigenic *E. coli* in fecal samples collected at week 4 post-weaning (data not shown).

**Table 1:** **Hematological traits of weanling pigs fed diets with vegetable oil or long-chain** ***omega*** **-3 fatty acids** ^**a**^

Trait	Experimental diets ^b^		***SEM***	***P*** ^***c***^
	Control	Omega		
WBC^d^ × 10^3^/mm^3^	14.2	15.7	1.2	0.44
Lymphocytes, %	42.3	41.5	4.0	0.90
Neutrophils, %	52.8	51.0	3.8	0.75
Eosinophils, %	1.5	2.8	0.4	0.07
Monocytes, %	2.5	4.3	0.7	0.13
RBC^e^ × 10^3^/mm^3^	7.0	6.6	0.3	0.45
Hemoglobin	10.4	9.2	0.7	0.28
Hematocrit, %	34.1	31.4	2.1	0.41
Platelets × 10^3^/mm^3^	378.5	674.0	80.4	0.04

^a^Means represent 4 pigs per dietary treatment.

^b^Diets were: Control (3% vegetable oil) and *omega* (3% Gromega Ultra 345, provided by JBS United, Inc., Sheridan, IN).

^c^ *P*-values for control compared to Omega diet.

^d^White blood cells.

^e^Red blood cells.

## Conclusions

In the pig, the period following weaning is generally characterized by sub-optimal growth, deteriorated feed efficiency, and a high incidence of diarrhea. Results of this study provided no evidence for *n*-3 PUFA modulation of growth of male weanling pigs raised in the absence of significant immunological and environmental challenges. The observation that female piglets consuming the *n*-3 PUFA-supplemented diet were lighter at week 4 post-weaning than those consuming the vegetable oil-enriched diet (Figure 2) may be indicative of a decrease in fat accretion at the expense of lean tissue. Additionally, dietary *n*-3 PUFA may improve the immune status of weanling pigs, as reflected by considerably lower plasma TNF-α in pigs consuming *n*-3 PUFA than those fed vegetable oil. The gradual increase in body weight, feed intake and feed efficiency following weaning likely reflects a progressive adaptation to post-weaning diets and a gradual improvement of the gastrointestinal microbiota.

## Methods

### Animals, diets and experimental design

The animal protocol for this research was approved by the institutional Animal Research Committee of the University of Florida. To avoid potential differences due to farrowing season, the study was conducted using 40 piglets born within one week at the Swine Research Unit of the University of Florida (Gainesville, FL) during the month of March 2013. Forty crossbred pigs (averaging 27 ± 2 days of age and 8.1 ± 0.7 kg of body weight) were balanced for initial body weight and gender across two treatment groups in a complete randomized block design. Experimental animals were fed either a control (3% vegetable oil, n = 20) or *n*-3 PUFA (3% marine *n*-3 PUFA; *Gromega 345*, JBS United, Inc, Sheridan IN, n = 20)-supplemented diet for four weeks after weaning. The vegetable oil was purchased from Sysco Corporations (Houston, TX) and contained approximately 22% total fat. Omega-3 fatty polyunsaturated fatty acids used in this study were provided by JBS United (Sheridan, Indiana) and contained a minimum of 39% crude fat. Complete ingredient compositions and FA profiles of experimental diets are summarized in Tables 2 and 3, respectively. Pigs were housed in pens (groups of 5 animals per pen; pen size = 2.4 m × 1.8 m) and kept on the same diet for the entire experimental period. Body weight and feed consumption were recorded weekly throughout the 4-week experiment. These observations were used to calculate ADG, ADFI, and G: F.

**Table 2:** **Ingredient and calculated compositions of experimental diets**

Composition	Experimental diets ^a^	Omega
	Control	
*Ingredient:*		
Corn, %	61.90	61.90
Soybean meal, %	25.00	25.00
Vegetable oil, %	3.00	-
Gromega Ultra 345, %	-	3.00
Min-Vit Premix, %	10.00	10.00
L-Lysine.HCL, %	0.10	0.10
*Calculated composition:*		
ME, kcal/kg	3282.38	3282.38
CP, %	19.53	19.53
CF, %	3.39	3.39
Lysine, %	1.40	1.40
Calcium, %	0.78	0.97
Phosphorus, %	0.63	0.63

^a^Diets were: Control (3% vegetable oil) and omega (3% Gromega Ultra 345, provided by JBS United, Inc., Sheridan, IN).

**Table 3:** **Fatty acid profile (g/100 g of total fat) of experimental diets** ^**a**^

Fatty acid	Experimental diets ^b^	***Omega***
	Control	
C14:0	0.21	2.61
C15:0	0.00	2.61
C16:0	14.68	19.76
C16:1, 9c	0.32	2.98
C17:0	0.13	0.42
C17:1	0.00	0.39
C18:0	4.26	4.71
C18:1, 9c	24.72	23.89
C18:2*n*-6	49.79	37.04
C18:3*n*-3	4.53	2.15
C18:4*n*-3	0.00	0.47
C20:0	0.38	0.40
C20:1*n*-9	0.00	0.71
C20:5*n*3	0.00	1.30
C22:0	0.41	0.26
C22:5*n*-3	0.00	0.26
C22:6*n*-3	0.00	0.96
C24:0	0.26	0.34
∑ *n*-6	49.79	37.04
∑ *n*-3	4.53	5.54
∑*n-*6 /∑*n*-3	10.99	6.69
∑ SFA	20.33	28.79
∑ UFA	79.36	70.15

^a^Fatty acid analysis was performed by the University of Missouri Analytical Laboratory.

^b^Diets were: Control (3% vegetable oil) and *omega* (3% Gromega Ultra 345, provided by JBS United, Inc., Sheridan, IN).

### Blood collection and analysis

On days 0, 14 and 28 of the experiment, jugular venous blood samples (8 ml from each experimental pig) were collected into evacuated heparinized tubes (BD Franklin Lakes, NJ) and centrifuged (3,000 × *g* for 15 min) to separate plasma. The plasma samples were stored at −80°C until analysis. Concentrations of IGF-I and TNF-α in plasma were analyzed using commercially available ELISA kits (R&D Systems, Inc., Minneapolis, MN). Hormone and cytokine analyses were performed in single assays and intra-assay CV were 4.0 and 4.7% for IGF-I and TNF-α, respectively. The least detectable concentrations were 0.06 ng/mL and 5.50 pg/ml. On day 27 of the experiment, additional blood samples were collected for complete blood cell counts, and hematological traits were determined as described by Quiroz-Rocha et al. [28].

### Fecal evaluation

Two fecal consistency scores were assigned to each pen on weeks 1, 2, 3, and 4 post-weaning. The scale used to assess fecal consistency was based on a numerical scale of 1 to 3, where 1 represented a normal (hard) feces, 2 represented a soft (moist) feces, and 3 represented diarrhea (watery liquid). The weekly score for each pen was calculated by averaging the two fecal consistency scores (Table 4).

**Table 4:** **Fecal consistency scores** ^**a**^ **of weanling pigs fed diets with vegetable oil or long-chain** ***omega*** **-3 fatty acids** ^**b**^

Week post-weaning	Diets ^c^	Omega	***SEM***	***P*** ^***d***^
	Control			
1	2.6	2.5	0.2	0.67
2	1.9	1.9	0.2	1.00
3	2.0	1.9	0.2	0.68
4	1.3	1.1	0.2	0.68

^a^The scale used for assessing fecal consistency was based on a numerical scale of 1 to 3, where 1 represented a normal (hard) feces, 2 represented a soft moist feces, and 3 represented diarrhea (watery liquid).

^b^Means represent average fecal scores for 4 pens per dietary treatment.

^c^Diets were: Control (3% vegetable oil) and *omega* (3% Gromega Ultra 345, provided by JBS United, Inc., Sheridan, IN).

^d^ *P*-values for control compared to *Omega* diet.

### Statistical analysis

Effects of diets on growth, IGF-I, TNF-α and fecal characteristics were analyzed using the MIXED procedure of Statistical Analysis System (version 9.3) with repeated measures [29]. For individual measurements (body weights), fixed effects included diet, gender, diet × gender interaction, week after weaning, diet × week interaction, gender × week interaction and diet × gender × week interaction. The pig, nested within gender and diet, was considered a random variable, and therefore the pig variance was used to test the effects of diet, gender, and diet x gender interaction. Initial weights were used as covariates in these analyses. A similar model was used to test the effect of diet on plasma IGF-I and TNF-α concentrations, except that week after weaning was replaced by day of blood sample collection. For collective measurements (feed intake, average daily gain, feed efficiency, and fecal consistency score), the statistical model included the effect of diets, pen (diet), week relative to weaning, diet × week interaction. In these models, pen was used as experimental unit to test the main effect of diet. Single blood samples were collected for complete blood cell counts, and, therefore, the statistical models for hematological traits contained only the main effect of diet. For all responses, significant differences between means were declared at *P* < 0.05.

## Abbreviations

ADFI: Average daily feed intake
ADG: Average daily gain
G: F: Gain to feed ratio
GH: Growth hormone
IGF-I: Insulin-like growth factor I
PPAR: Peroxisome proliferator-activated receptors
RBC: Red blood cells
TNF-α: Tumor necrosis factor alpha
WBC: White blood cells.

## References

[1] Le Dividich J, Sève B (2000). Effects of underfeeding during the weaning period on growth, metabolism, and hormonal adjustments in the piglet. Domest Anim Endocrinol.

[2] Montagne L, Boudry G, Favier C, Le Huërou-Luron I, Lallès JP, Sève B (2007). Main intestinal markers associated with the changes in gut architecture and function in piglets after weaning. Br J Nutr.

[3] Pié S, Lallès JP, Blazy F, Laffitte J, Sève B, Oswald IP (2004). Weaning is associated with an up-regulation of expression of inflammatory cytokines in the intestine of piglets. J Nutr.

[4] Cromwell GL (2002). Why and how antibiotics are used in swine production. Anim Biotechnol.

[5] Vondruskova H, Slamova R, Trckova M, Zraly Z, Pavlik I (2010). Alternatives to antibiotic growth promoters in prevention of diarrhoea in weaned piglets: a review. Vet Medic.

[6] Liu YL, Li DF, Gong LM, Yi GF, Gaines AM, Carroll JA (2003). Effects of fish oil supplementation on the performance and immunological, adrenal, and somatotropic responses of weaned pigs after an Escherichia coli lipopolysaccharide challenge. J Anim Sci.

[7] Gabler NK, Radcliffe JS, Spencer JD, Webel DM, Spurlock ME (2009). Feeding long-chain *n*-3 polyunsaturated fatty acids during gestation increases intestinal glucose absorption potentially via the acute activation of AMPK. J Nutr Biochem.

[8] Heo JM, Opapeju FO, Pluske JR, Kim JC, Hampson DJ, Nyachoti CM (2012). Gastrointestinal health and function in weaned pigs: a review of feeding strategies to control post-weaning diarrhoea without using in-feed antimicrobial compounds. J Anim Physiol Anim Nutr.

[9] Calder PC (2010). Omega-3 fatty acids and inflammatory processes. Nutrients.

[10] Calder PC (2012). Omega-3 polyunsaturated fatty acids and inflammatory processes: nutrition or pharmacology?. Br J Clin Pharmacol.

[11] Carroll JA, Gaines AM, Spencer JD, Allee GL, Kattesh HG, Roberts MP, Zannelli ME (2003). Effect of menhaden fish oil supplementation and lipopolysaccharide exposure on nursery pigs. I. Effects on the immune axis when fed diets containing spray-dried plasma. Domest Anim Endocrinol.

[12] Korver DR, Klasing KC (1997). Dietary fish oil alters specific inflammatory immune responses in chicks. J Nutr.

[13] Eastwood L, Kish PR, Beaulieu AD, Leterme P (2009). Nutritional value of flaxseed meal for swine and its effects on the fatty acid profile of the carcass. J Anim Sci.

[14] Baillie RA, Takada R, Nakamura M, Clarke SD (1999). Coordinate induction of peroxisomal acyl-CoA oxidase and UCP-3 by dietary fish oil: a mechanism for decreased body fat deposition. Prostaglandins Leukot Essent Fatty Acids.

[15] Belzung F, Raclot T, Groscolas R (1993). Fish oil n-3 fatty acids selectively limit the hypertrophy of abdominal fat depots in growing rats fed high-fat diets. Am J Physiol.

[16] Hill JO, Peters JC, Lin D, Yakubu F, Greene H, Swift L (1993). Lipid accumulation and body fat distribution is influenced by type of dietary fat fed to rats. Int J Obes Relat Metab Disord.

[17] Jump DB, Clark SD, Thelen A, Liimatta M (1994). Coordinate regulation of glycolytic and lipogenic gene expression by polyunsaturated fatty acids. J Lipid Res.

[18] Noreen EE, Sass MJ, Crowe ML, Pabon VA, Brandauer J, Averill LK (2010). Effects of supplemental fish oil on resting metabolic rate, body composition, and salivary cortisol in healthy adults. J Int Soc Sports Nutr.

[19] Thissen JP, Verniers J (1997). Inhibition by interleukin-1β and tumor necrosis factor-α of the insulin-like growth factor I messenger ribonucleic acid response to growth hormone in rat hepatocyte primary culture. Endocrinology.

[20] Bemelmans MH, van Tits LJ, Buurman WA (1996). Tumor necrosis factor: Function, release and clearance. Crit Rev Immunol.

[21] Lo CJ, Chiu KC, Fu M, Lo R, Helton S (1999). Fish oil decreases tumor necrosis factor gene transcription by altering the NF-κΒ activity. J Surg Res.

[22] Novak TE, Babcock TA, Jho DH, Helton WS, Espat NJ (2003). NF-κB inhibition by ώ-3 fatty acids modulates LPS-stimulated macrophage TNF-α transcription. Am J Physiol Lung Cell Mol Physiol.

[23] Zhao Y, Joshi-Barve S, Barve S, Chen LH (2004). Eicosapentaenoic acid prevents LPS-induced TNF-α expression by preventing NF-kB activation. J Am Coll Nutr.

[24] Gaines AM, Carroll JA, Yi GF, Allee GL, Zannelli ME (2003). Effect of menhaden fish oil supplementation and lipopolysaccharide exposure on nursery pigs. II. Effects on the immune axis when fed simple or complex diets containing no spray-dried plasma. Domest Anim Endocrinol.

[25] Malekshahi Moghadam A, Saedisomeolia A, Djalali M, Djazayery A, Pooya S, Sojoudi F (2012). Efficacy of omega-3 fatty acid supplementation on serum levels of tumour necrosis factor-alpha, C-reactive protein and interleukin-2 in type 2 diabetes mellitus patients. Singapore Med J.

[26] Friendship RM, Lumsden JH, McMillan I, Wilson MR (1984). Hematology and biochemistry reference values for Ontario swine. Can J Comp Med.

[27] Wilson GD, Harvey DG, Snook CR (1972). A review of factors affecting blood biochemistry in the pig. Br Vet J.

[28] Quiroz-Rocha GF, LeBlanc SJ, Duffield TF, Wood D, Leslie KE, Jacobs RM (2009). Reference limits for biological and hematological analytes of dairy cows one week before and one week after parturition. Can Vet J.

[29] Littell RC, Henry PR, Ammerman CB (1998). Statistical analysis of repeated measures data using SAS procedures. J Anim Sci.

